# Supplementary material for: Voxelated bioprinting of modular double-network bio-ink droplets
Source: Nat Commun. 2024 Jul 13;15:5902. doi: 10.1038/s41467-024-49705-z (PMC11246467; doi:10.1038/s41467-024-49705-z)
Supplement: Supplementary file 1 — Supplementary Information [file 41467_2024_49705_MOESM1_ESM.pdf]

Supplementary Information for

Voxelated bioprinting of modular double-network bio-ink droplets

Jinchang Zhu,[1] Yi He,[2] Yong Wang,[2] Li-Heng Cai[1,3,4*]

Affiliations:

[1]Soft Biomatter Laboratory, Department of Materials Science and Engineering, University of Virginia, Charlottesville, VA 22904, USA

[2]Department of Surgery, University of Virginia, Charlottesville, VA 22903, USA

[3]Department of Chemical Engineering, University of Virginia, Charlottesville, VA 22904, USA

[4]Department of Biomedical Engineering, University of Virginia, Charlottesville, VA 22904, USA

*Corresponding author. Email: liheng.cai@virginia.edu

The PDF file includes:

Supplementary Figs. 1 to 16

Other Supplementary Information for this manuscript include the following:

Supplementary Movies 1 to 7

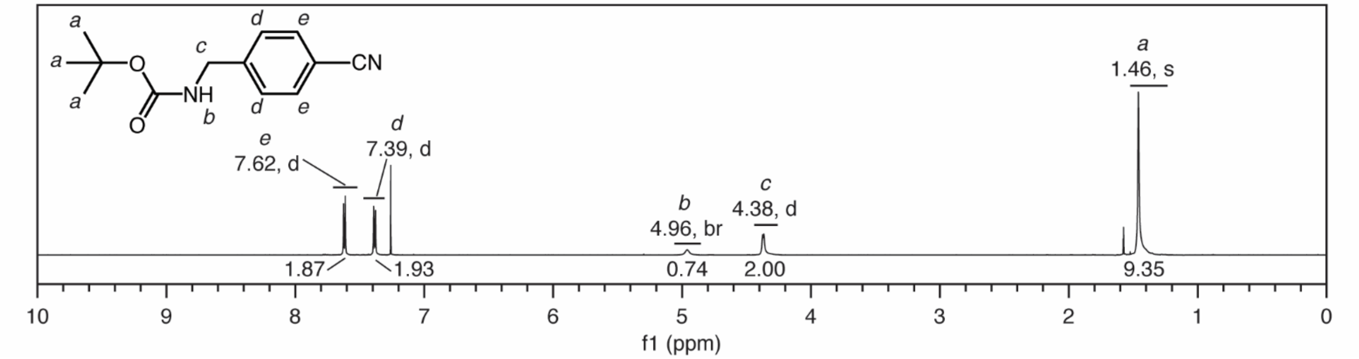

Supplementary Fig. 1. ^{1}H NMR spectrum of *tert*-butyl *N*-(4-cyanobenzyl)carbamate.

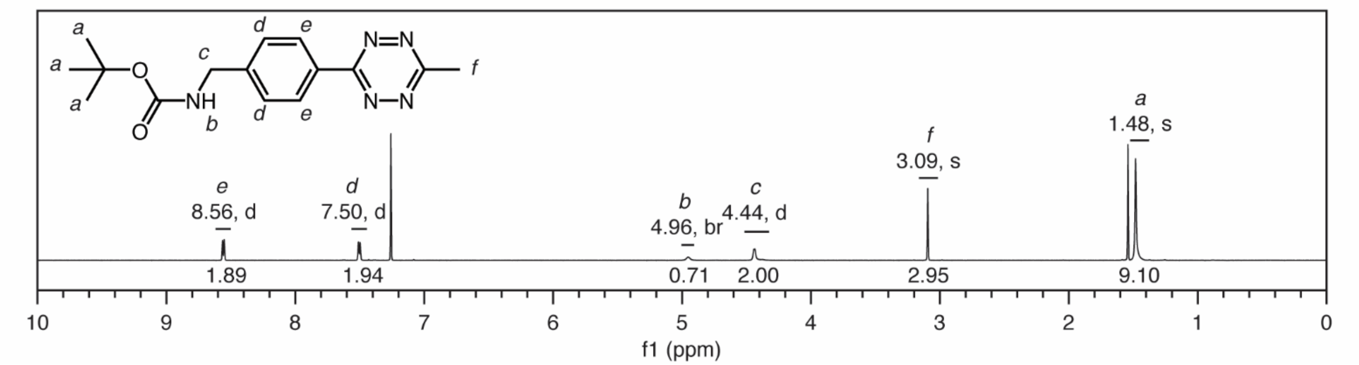

Supplementary Fig. 2. ^{1}H NMR of *tert*-butyl *N*-[4-(6-methyl-1,2,4,5-tetrazin-3-yl)benzyl]carbamate.

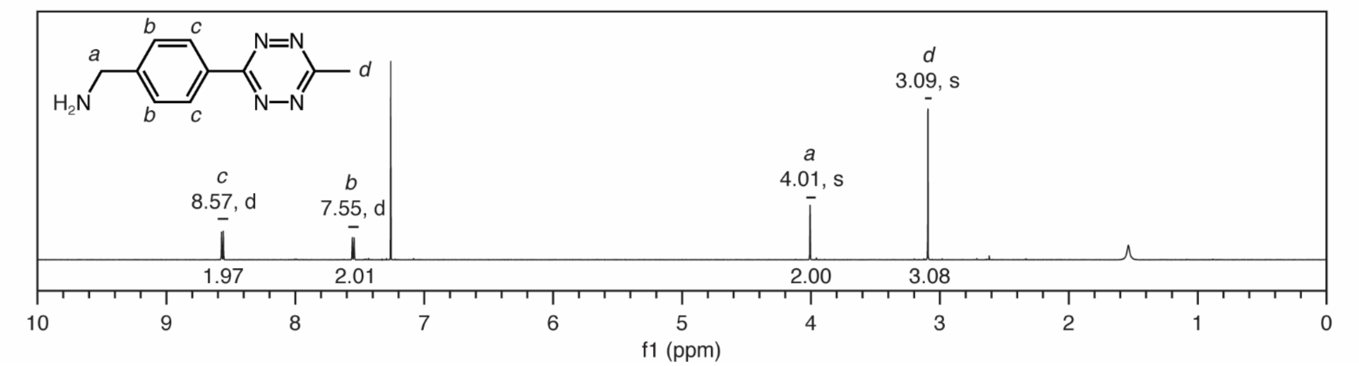

Supplementary Fig. 3. ^{1}H NMR of [4-(6-methyl-1,2,4,5-tetrazin-3-yl) phenyl]methanamine.

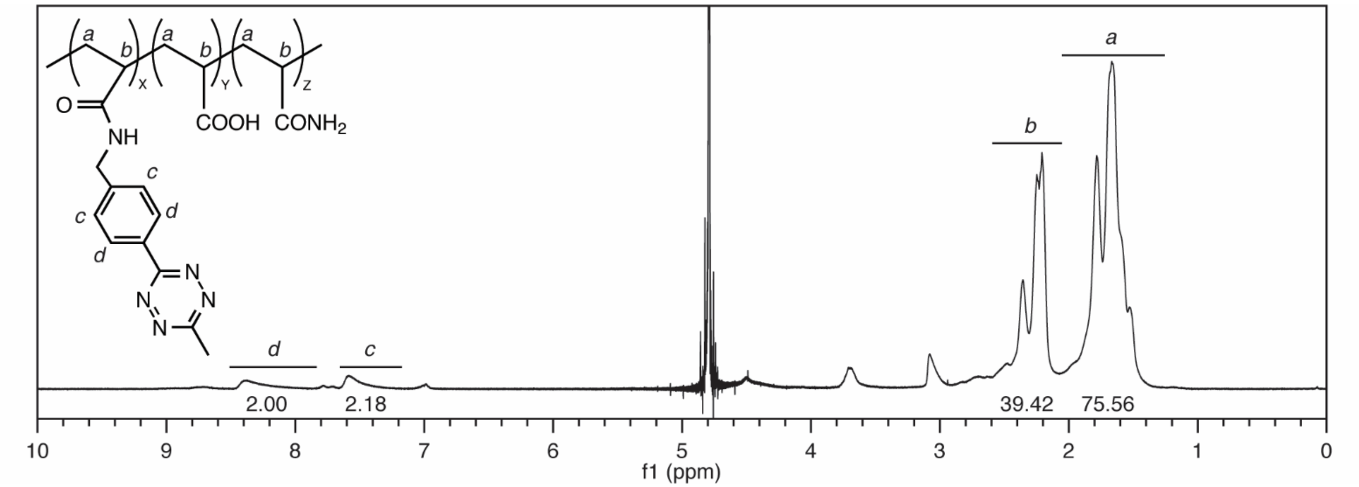

Supplementary Fig. 4. ^{1}H NMR of tetrazine modified poly(acrylamide-*co*-acrylic acid).

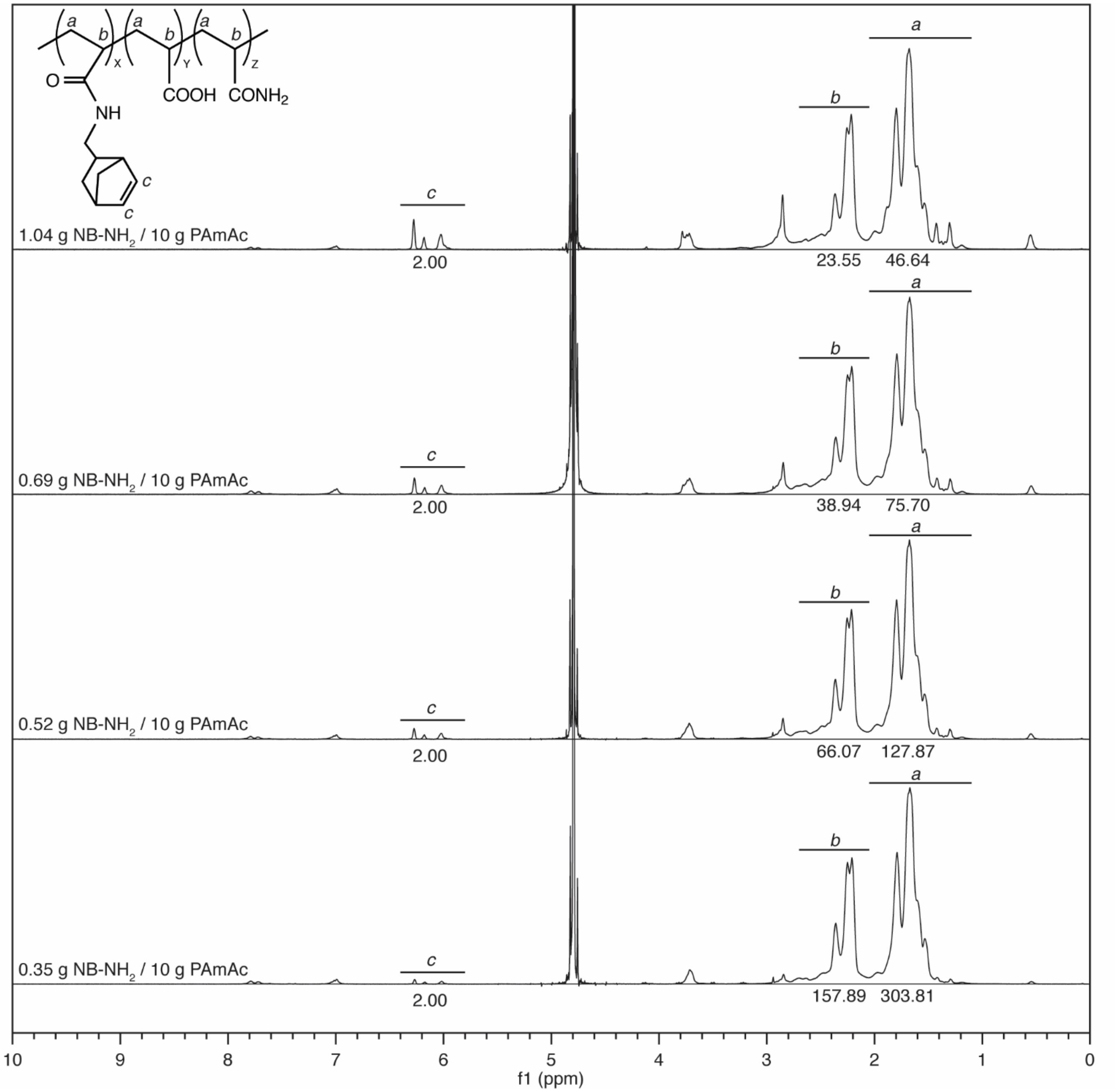

Supplementary Fig. 5. ^{1}H NMR of norbornene modified poly(acrylamide-*co*-acrylic acid).

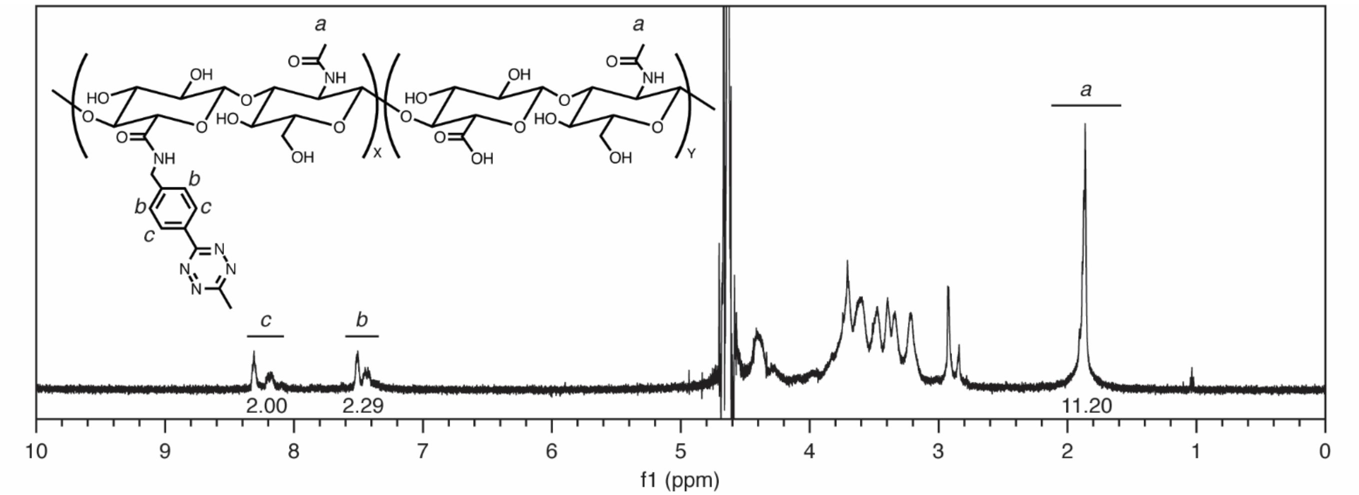

Supplementary Fig. 6. ^{1}H NMR of tetrazine modified hyaluronic acid.

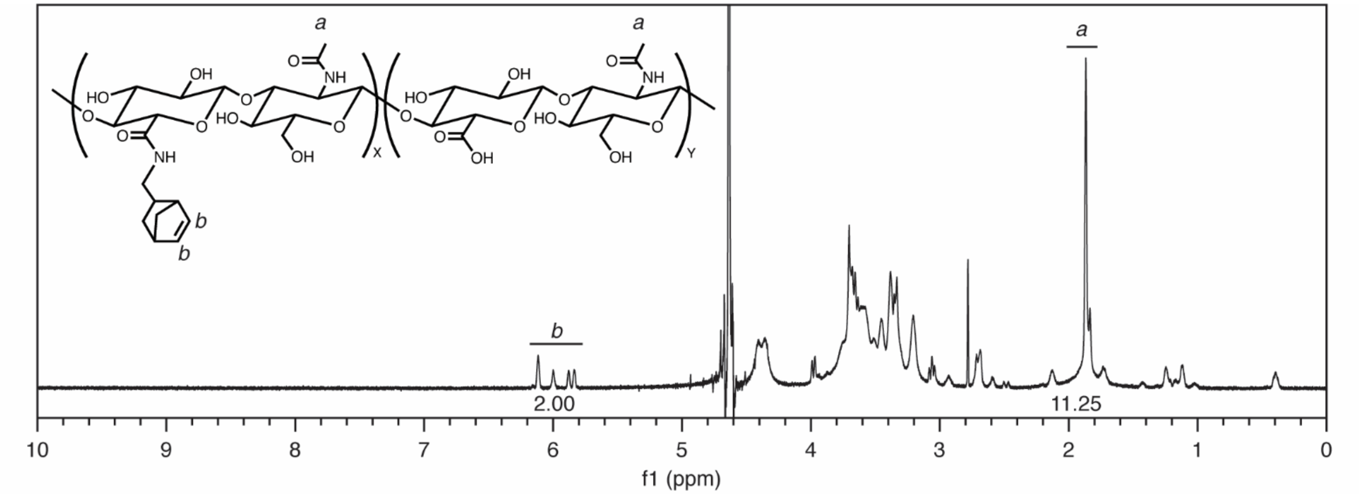

Supplementary Fig. 7. ^{1}H NMR of norbornene modified hyaluronic acid.

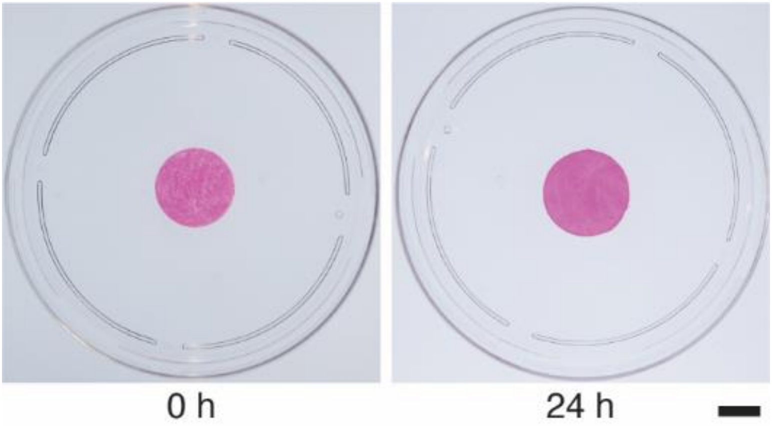

Supplementary Fig. 8. Swelling of a bulk DN hydrogel. Photos of the DN $PAM_{10}Alg_2$ hydrogel in DMEM media up to 24h. Scale bar, 10 mm.

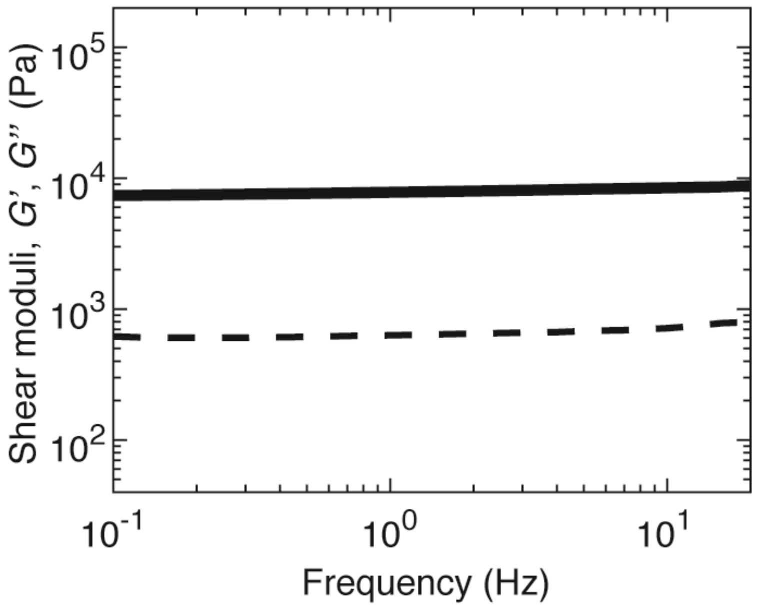

Supplementary Fig. 9. Dynamic mechanical properties of a double-network hydrogel. Dependencies of storage (G', solid line) and loss (G'', dashed line) moduli of completely crosslinked double-network $\mathrm{PAM}_{10}^{1/4}\mathrm{Alg}_2$ with a mismatched TZ and NB grafting ratio. $\mathrm{PAM}_{10}^{1/4}\mathrm{Alg}_2$ is made from TZ-PAM with a grafting ratio of 2.53% and NB-PAM with a grafting ratio of 0.63%. The composition of a crosslinked hydrogel is denoted as $\mathrm{PAM}_x^z\mathrm{Alg}_y$, where x is the concentration in (w/v)% for PAM consisting of equal amount of TZ-PAM and NB-PAM, y is the concentration in (w/v)% for alginate, and z is the molar ratio between NB and TZ groups.

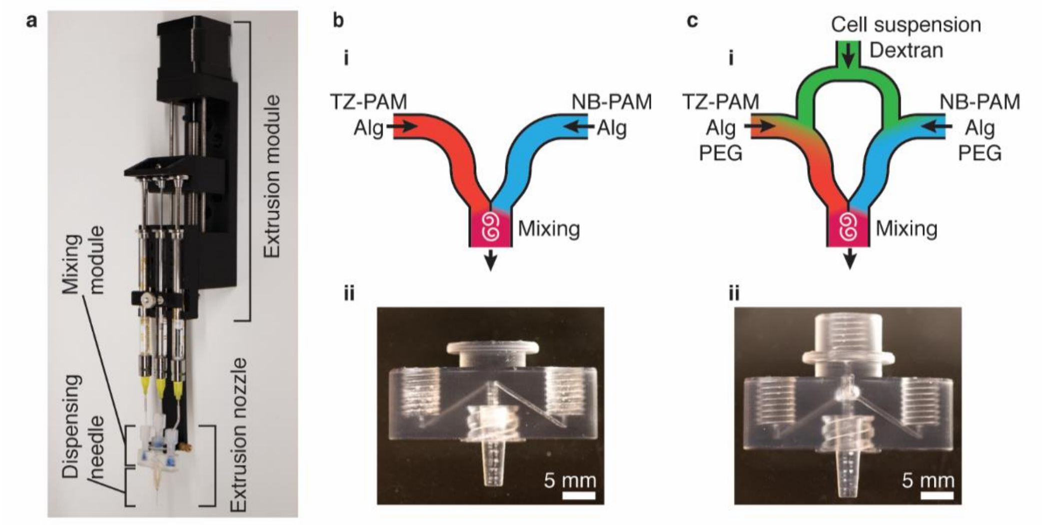

Supplementary Fig. 10. Setup of the DASP 2.0. (**a**) Assembly of the extrusion module, syringes, and extrusion nozzle. (**b**) (i) Schematic and (ii) photograph of the dual-inlet mixing module. (**c**) (i) Schematic and (ii) photograph of the triple-inlet mixing module.

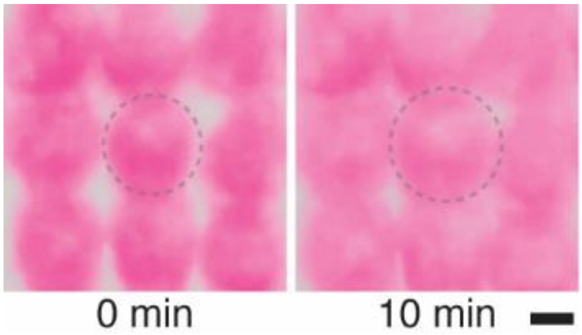

Supplementary Fig. 11. Swelling of DASP printed DN bio-ink droplets. Photos of DASP printed DN bio-ink droplets in the supporting matrix until equilibrium. The droplets are made of made of DN $PAM_{10}Alg_2$ hydrogel. Scale bar, 500 μm.

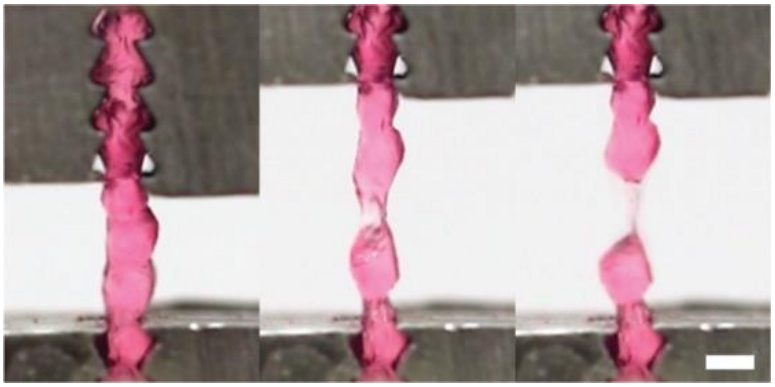

Supplementary Fig. 12. Snapshots of a DASP printed 1D filament under uniaxial elongation. The bio-ink is $PAM_{10}Alg_2$ and the tensile strain rate is 0.05/sec. The left, middle, and right panels are, respectively, captured at the beginning of the tensile test, right before breaking, and right after breaking. Scale bar, 1mm

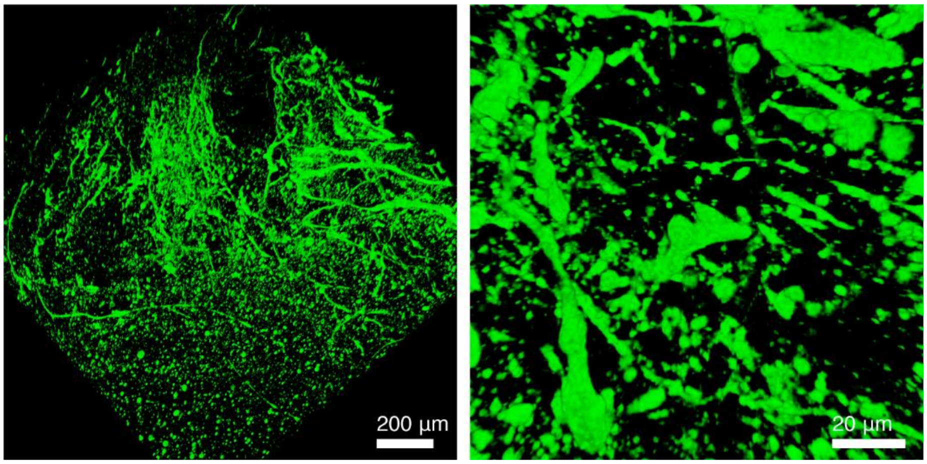

Supplementary Fig. 13. Microstructure of hydrogels formed by aqueous-two phase system. Fluorescence confocal microscopy images depicting the PEG/dextran aqueous two-phase system mixed by the extrusion nozzle. Green phase, dextran with a concentration of 15% (w/v) and FITC labeled dextran with a concentration of 0.2% (w/v). Dark phase: PEG with a concentration of 15% (w/v) and alginate with a concentration of 2% (w/v), which is crosslinked by 20 mM calcium ion. The images are generated via 3D reconstruction from multiple 2D images.

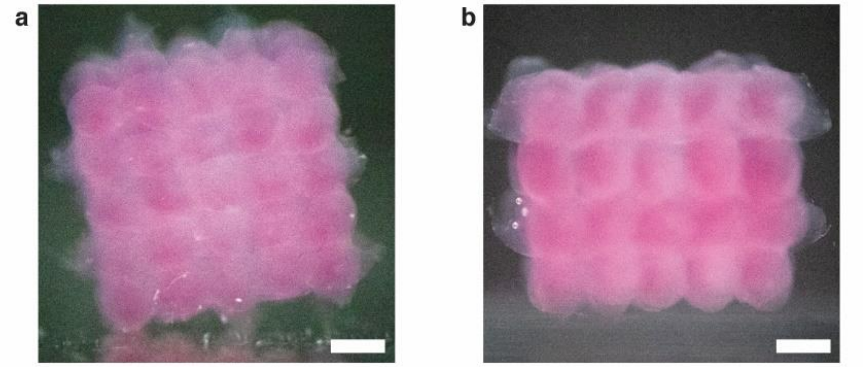

Supplementary Fig. 14. A DASP printed 3D lattice. The 5×5×4 lattice consists of 100 interconnected yet distinguishable DN $PAM_{10}Alg_2$ hydrogel particles. (**a**) and (**b**) display the top and lateral view, respectively. Scale bars, 1 mm.

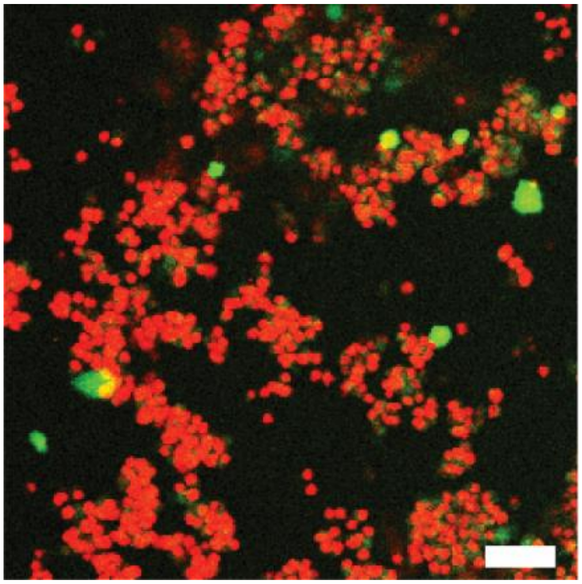

Supplementary Fig. 15. Cytocompatibility of free polyacrylamide polymers. A representative fluorescence confocal microscopy image from live/dead assay of Beta-TC-6 cells. The cells with a density of 2×10^{7}/mL are incubated in DMEM with 10% w/v polyacrylamide for 3h and subsequently suspended to cell culture media to reach a final density of 1.2×10^{5}/mL for cell recovery. The image is taken 12 h after the recovery. Scale bar, 50 μm.

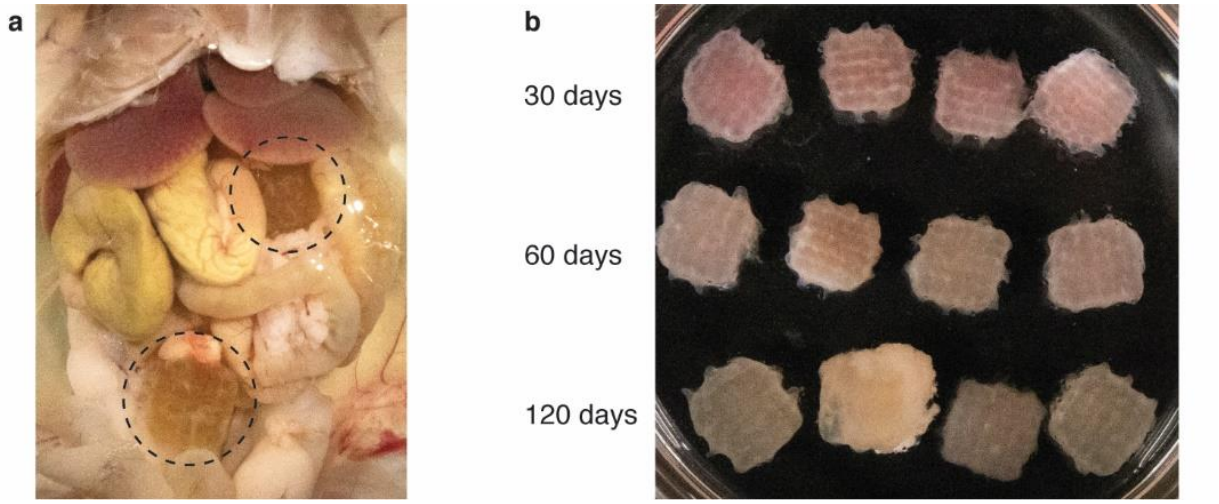

Supplementary Fig. 16. *In vivo* stability of DASP printed scaffolds. Representative photographs of the retrieved DN $PAM_{10}Alg_2$ scaffolds. (**a**) A photograph of the scaffolds located near the liver in the abdominal cavity (dashed circles). (**b**) A photograph of the all scaffolds retrieved at 30, 60 and 120 days after transplantation.
