# Supplementary material for: Voxelated bioprinting of modular double-network bio-ink droplets
Source: Nat Commun. 2024 Jul 13;15:5902. doi: 10.1038/s41467-024-49705-z (PMC11246467; doi:10.1038/s41467-024-49705-z)
Supplement: Supplementary file 3 — Description of Additional Supplementary Files [file 41467_2024_49705_MOESM3_ESM.pdf]

Description of Additional Supplementary Files

File Name: Supplementary Movie 1
Description: Tensile tests of dog-bone shape samples of PAM-based double-network hydrogels.

File Name: Supplementary Movie 2
Description: Mixing a pair of viscoelastic bio-inks using a dual-inlet print nozzle.

File Name: Supplementary Movie 3
Description: Manipulation of a hollow sphere printed by DASP 2.0.

File Name: Supplementary Movie 4
Description: A DASP 2.0 printed hollow sphere cut into two pieces.

File Name: Supplementary Movie 5
Description: Manipulation of a DASP 2.0 printed gyroid made of double-network hydrogel.

File Name: Supplementary Movie 6
Description: Cyclic compression test of a gyroid made of double-network hydrogel.

File Name: Supplementary Movie 7
Description: Tensile test of a dog-bone shape sample of HA-based double-network hydrogel.
