# Supplementary material for: Voxelated bioprinting of modular double-network bio-ink droplets
Source: Nat Commun. 2024 Jul 13;15:5902. doi: 10.1038/s41467-024-49705-z (PMC11246467; doi:10.1038/s41467-024-49705-z)
Supplement: Supplementary file 11 — Reporting Summary [file 41467_2024_49705_MOESM11_ESM.pdf]

nature portfolio

Corresponding author(s): Liheng Cai

Last updated by author(s): Jun 10, 2024

Reporting Summary

Nature Portfolio wishes to improve the reproducibility of the work that we publish. This form provides structure for consistency and transparency in reporting. For further information on Nature Portfolio policies, see our Editorial Policies and the Editorial Policy Checklist.

Statistics

For all statistical analyses, confirm that the following items are present in the figure legend, table legend, main text, or Methods section.

n/a	Confirmed	
☐	☒	The exact sample size (n) for each experimental group/condition, given as a discrete number and unit of measurement
☐	☒	A statement on whether measurements were taken from distinct samples or whether the same sample was measured repeatedly
☒	☐	The statistical test(s) used AND whether they are one- or two-sided<br>*Only common tests should be described solely by name; describe more complex techniques in the Methods section.*
☒	☐	A description of all covariates tested
☒	☐	A description of any assumptions or corrections, such as tests of normality and adjustment for multiple comparisons
☐	☒	A full description of the statistical parameters including central tendency (e.g. means) or other basic estimates (e.g. regression coefficient) AND variation (e.g. standard deviation) or associated estimates of uncertainty (e.g. confidence intervals)
☒	☐	For null hypothesis testing, the test statistic (e.g. F, t, r) with confidence intervals, effect sizes, degrees of freedom and P value noted<br>*Give P values as exact values whenever suitable.*
☒	☐	For Bayesian analysis, information on the choice of priors and Markov chain Monte Carlo settings
☒	☐	For hierarchical and complex designs, identification of the appropriate level for tests and full reporting of outcomes
☒	☐	Estimates of effect sizes (e.g. Cohen's d, Pearson's r), indicating how they were calculated

Our web collection on statistics for biologists contains articles on many of the points above.

Software and code

Policy information about availability of computer code

Data collection	All data are collected using the software provided by the equipment manufacturer; these include NMR, rheometry, tensile and compression test, and confocal microscopy.
Data analysis	Data was analyzed using MATLAB for curve fitting and FIJI for image analysis.

For manuscripts utilizing custom algorithms or software that are central to the research but not yet described in published literature, software must be made available to editors and reviewers. We strongly encourage code deposition in a community repository (e.g. GitHub). See the Nature Portfolio guidelines for submitting code & software for further information.

Data

Policy information about availability of data

All manuscripts must include a data availability statement. This statement should provide the following information, where applicable:

- Accession codes, unique identifiers, or web links for publicly available datasets
- A description of any restrictions on data availability
- For clinical datasets or third party data, please ensure that the statement adheres to our policy

All data that support the findings of this study are included the manuscript and the Supplementary Materials. Raw data are provided with this paper.

Research involving human participants, their data, or biological material

Policy information about studies with human participants or human data. See also policy information about sex, gender (identity/presentation), and sexual orientation and race, ethnicity and racism.

Reporting on sex and gender	Not applicable.
Reporting on race, ethnicity, or other socially relevant groupings	Not applicable.
Population characteristics	Not applicable.
Recruitment	Not applicable.
Ethics oversight	Not applicable.

Note that full information on the approval of the study protocol must also be provided in the manuscript.

Field-specific reporting

Please select the one below that is the best fit for your research. If you are not sure, read the appropriate sections before making your selection.

☒ Life sciences ☐ Behavioural & social sciences ☐ Ecological, evolutionary & environmental sciences

For a reference copy of the document with all sections, see nature.com/documents/nr-reporting-summary-flat.pdf

Life sciences study design

All studies must disclose on these points even when the disclosure is negative.

Sample size	The sample size for cytocompatibility and GSIS tests is 5, which meets the requirement for statistical analysis that requires $n \geq 3$.
Data exclusions	No data exclusions.
Replication	For all experiments, at least three independent measurements were performed. All replication experiments are successful.
Randomization	Data were not randomized. For imaging, random fields of view were selected.
Blinding	Blinding is not applicable due to the obvious difference in sample appearance between the naked and encapsulated cells.

Reporting for specific materials, systems and methods

We require information from authors about some types of materials, experimental systems and methods used in many studies. Here, indicate whether each material, system or method listed is relevant to your study. If you are not sure if a list item applies to your research, read the appropriate section before selecting a response.

Materials & experimental systems

n/a	Involved in the study	
☒	☐	Antibodies
☐	☒	Eukaryotic cell lines
☒	☐	Palaeontology and archaeology
☐	☒	Animals and other organisms
☒	☐	Clinical data
☒	☐	Dual use research of concern
☒	☐	Plants

Methods

n/a	Involved in the study	
☒	☐	ChIP-seq
☒	☐	Flow cytometry
☒	☐	MRI-based neuroimaging

Eukaryotic cell lines

Policy information about cell lines and Sex and Gender in Research

Cell line source(s)	Beta-TC-6 cells are received as a gift from Dr. Yong Wang, which was purchased from the American Type Culture Collection (Cat. No. CRL-11506).
Authentication	Beta-TC-6 cells are certified by the supplier American Type Culture Collection.
Mycoplasma contamination	Cells are tested negative for mycoplasma contamination.
Commonly misidentified lines (See ICLAC register)	Not applicable.

Animals and other research organisms

Policy information about studies involving animals; ARRIVE guidelines recommended for reporting animal research, and Sex and Gender in Research

Laboratory animals	C57BL/6 mouse (male, 12 weeks old, Jackson Laboratory). Animals are housed in cages supplied with food pile, water, and corncob bedding. The cages are located in an animal facility with a 12-h light/12-h dark cycle, a temperature of 22 °c, and a relative humidity of 50%.
Wild animals	Not applicable.
Reporting on sex	The animals studies are performed only on male C57BL/6 mice because comparing with female mice, male mice are more active in body movement, creating vigorous mechanical perturbation in the abdominal cavity for testing in vivo stability of 3D printed scaffolds.
Field-collected samples	Not applicable.
Ethics oversight	The animal studies are performed following a protocol approved by the University of Virginia Animal Care and Use Committee (Protocol No. 4360-08-21).

Note that full information on the approval of the study protocol must also be provided in the manuscript.

Plants

Seed stocks	Not applicable.
Novel plant genotypes	Not applicable.
Authentication	Not applicable.
